# Effectiveness of single-session therapy for adult common mental disorders: a systematic review

**DOI:** 10.1186/s40359-023-01410-0

**Published:** 2023-11-07

**Authors:** Jongtae Kim, Namgil Ryu, Dixon Chibanda

**Affiliations:** 1 Department of Psychiatry, College of Medicine, The Catholic University of Korea, Seoul, Republic of Korea; 2 Department of Psychiatry, Seoul St. Mary’s Hospital, College of Medicine, The Catholic University of Korea, Seoul, Republic of Korea; 3 Department of Population Health, Faculty of Epidemiology and Population Health, London School of Hygiene and Tropical Medicine, London, UK

**Keywords:** Common mental disorder, Adult, Single-session therapy, Treatment gap, Systematic review

## Abstract

**Background:**

Common mental disorders (CMDs) impose significant socioeconomic impacts on the global community. Nevertheless, over 50% of individuals with CMDs do not receive proper treatment, indicating that the current treatment modalities do not adequately tackle this issue. Since single-session therapy (SST) is a potential method for reducing the treatment gap, it is crucial to evaluate its effectiveness. Therefore, this systematic review aimed to evaluate the effectiveness of SST on CMD symptoms in adults.

**Methods:**

This systematic review included randomised and non-randomised studies assessing the clinical effectiveness of SST on CMD symptoms in adults. English-written, peer-reviewed studies or dissertations were included, while grey literature was excluded. MEDLINE, Embase, PsycINFO, and Cochrane’s CENTRAL were searched on December 13, 2022, from their inception dates. The risk of bias in the included studies was evaluated using RoB 2 and ROBINS-I. A narrative synthesis was performed. This systematic review was registered in the PROSPERO database on July 6, 2022 (CRD42022343925).

**Results:**

Six randomised and three non-randomised studies were included after screening 2,130 records. Three non-randomised studies with a “critical” or “serious” risk of bias were excluded from the synthesis. Therefore, six randomised trials involving 298 participants were included in the synthesis. Four out of six studies had a “high” risk of bias. The participants had non-severe symptoms at baseline, and three intervention types (behavioural activation, DBT, and solution-focused psychotherapy) were evaluated. Five of six studies showed positive results for depression, with only one reporting a positive result for anxiety.

**Conclusions:**

SST may be effective in improving CMD symptoms in adults, particularly depression. However, there is a limit to deriving definite conclusions due to a high risk of bias in included studies, insufficient sample size and research volume. Further research exploring the characteristics of clients who can benefit from SST is required to facilitate its widespread use.

**Supplementary Information:**

The online version contains supplementary material available at 10.1186/s40359-023-01410-0.

## Background

Common mental disorders (CMD) are defined as a group of disorders with depressed mood or anxiety as the main symptoms, commonly seen in primary care or community settings [1]. Official diagnostic criteria, such as the Diagnostic and Statistical Manual of Mental Disorders, Fifth Edition (DMS-5) [2], distinguish between depression and anxiety disorders. Nonetheless, the concept of CMD encompassing both is legitimate from a public health standpoint, as they frequently co-occur and have similar treatment responses and epidemiological characteristics [1, 3–6].

Globally, CMDs impose a substantial socioeconomic impact. In 2015, depression was estimated to affect 322 million individuals of the global population [7], an increase of 18.4% compared to 2005 [8]. Years Lived with Disability (YLD) can be used to quantify the health and functioning loss caused by CMDs [7]. In 2015, depression contributed to more than 50 million YLD worldwide, indicating that depression is the single largest cause of YLD during that period [7]. Anxiety disorder was estimated to affect 264 million individuals in 2015 [7], an increase of 14.9% compared to 2005 [8]. It was the sixth-largest cause of YLD in 2015 [7]. Depression and anxiety disorder cost the global economy $1 trillion yearly in lost productivity [9].

Despite the growing global burden of CMDs, the treatment gap is remarkable. The treatment gap refers to the proportion of people who need care for a given condition but are left untreated [10]. It is reported that over 50% of those with CMDs do not obtain timely and proper care worldwide [10–12]. This treatment gap tends to be more pronounced in low-resource settings [13], and insufficient investment in mental health systems is one of the contributing factors. In low-income countries, only about 1% of national health expenditures are allocated to mental health sectors [14]. The number of mental health workers is only one-sixtieth that of high-income countries [14]. Given that there is not much substantial variation in the prevalence of CMDs around the globe [7], these inadequate mental health resources make it hard to meet treatment demands compared to nations with adequate resources. In addition, the treatment gap is exacerbated by decreased demand [13]. In a low-resource setting, there is evidence that persons with CMDs view their emotional difficulties as a result of social adversity rather than a condition requiring psychological or psychiatric treatment, resulting in low demand for mental health services [13]. They tend to question the utility of the services unless they see improvements in their social or economic conditions [13].

Psychotherapy is an important therapeutic option for CMDs [15, 16]. However, its conventional multi-session format can aggravate the treatment gap for two reasons. First, multi-session inevitably results in lengthy waiting times in a context with a high demand for mental health services, making timely treatment challenging. Second, it is difficult to meet the multi-session therapy assumption that clients will attend all planned sessions, particularly in low-resource environments where emotional suffering is perceived as a result of social determinants [13]. They tend to drop out early because they do not believe the treatment will benefit them if socioeconomic hardship continues [13].

Integrating single-session therapy (SST) into existing mental health services could reduce the treatment gap by addressing the abovementioned issues. SST can be defined as organised programmes intended to be provided in a single session, where practically any therapeutic approach could be applied [17, 18]. The main characteristic is that each session is viewed as self-contained by both the client and counsellor [19]. SST tends to be pragmatic, focusing on the current difficulty rather than its underlying causes [20]. Since SST is a self-contained intervention that does not expect the client to return after a single session, it could reduce the treatment gap caused by the client dropping out of treatment during multi-session therapy. In other words, while SST may not address the treatment gap for individuals who never seek mental health services in the first place, it can be effective in mitigating the treatment gap resulting from early dropouts by ensuring that those seeking help receive a pragmatic intervention promptly. Furthermore, the brevity of its format could shorten the waiting time, allowing individuals needing treatment to receive timely intervention.

The rationale of SST is based on findings indicating that the most common frequency of therapeutic sessions people attend is one and that the vast majority (68–88%) of those who did not attend after the first session were satisfied with that session [21]. In 2001, Bloom published a review that provided a summary of studies evaluating the effectiveness of SST [22]. While uncontrolled studies indicated promising results, Bloom (2001) noted the dearth of controlled studies [22]. In a subsequent review, Hurn (2005) stated that, despite the considerable potential of SST, there was a shortage of scientific evidence [23]. Campbell's (2012) review indicated that research had not improved much in terms of methodological rigour since Bloom's (2001) review [18]. Hymmen et al. (2013) identified key limitations in the published literature, including the lack of randomised controlled trials, the underuse of standardised clinical outcome measures, the small sample size, the homogeneous sample, and the bias resulting from the method of collecting outcome data [24]. Schleider & Weisz (2017) conducted a meta-analysis of randomised controlled trials and concluded that SST for youth mental health problems has a significant positive effect with an overall small-to-medium effect size [25]. A systematic review of randomised controlled studies undertaken by Bertuzzi et al. (2021) reported that SST is more effective than no treatment for reducing anxiety in youth and adults [26]. However, most of the included research focused on narrowly defined conditions, such as a specific phobia [26].

To the best of our current knowledge, no review has yet investigated the effectiveness of SST on adults’ CMD symptoms. It is indicative of the severity of CMDs in the adult population that depression and anxiety disorders are the leading and second-leading causes of YLD among adults of all ages [27]. Embedding SST into primary care or community settings where individuals with CMDs are frequently seen could be a potential model for reducing this massive burden by addressing the treatment gap. To do this, it is necessary to generate evidence of its clinical benefit. Therefore, this systematic review aims to assess the clinical effectiveness of SST against various control conditions, including no control, in addressing CMD symptoms in adults.

## Methods

This systematic review was registered on the PROSPERO database on July 6, 2022 (registration number: CRD42022343925) and followed the Preferred Reporting Items for Systematic Reviews and Meta-Analyses (PRISMA) 2020 statement [28].

## Eligibility criteria

### Participants

This review included studies targeting adults with depressive or anxiety symptoms commonly seen in primary care or community-based settings. This review included studies in which participants met official diagnostic criteria (e.g., DSM-5). Additionally, studies with participants who did not meet the diagnostic criteria were included if their pre- and post-intervention CMD symptoms were reported. This is because the primary goal of this review was to assess the effectiveness of SST on symptom reduction rather than complete remission of disorders. Studies involving participants under 18 or without demographic information were excluded.

### Intervention

This review included studies focused on interventions initially designed to be provided in one session by the same therapist with no extra intervention (e.g., reminder emails). Studies focused on non-in-person based (e.g., online-based), computer-assisted, self-guided, and non-talk-based (e.g., meditation or exercise) interventions were excluded. Studies evaluating group or family therapy were excluded. In addition, studies focused on specific interventions addressing narrowly defined symptoms that would not typically be dealt with in primary care or community-based services (e.g., exposure therapy for specific phobia [29] or cognitive behavioural education for intrusive memories [30]) were excluded.

### Control

This review did not place any limitations on the control conditions. Therefore, including those without a control arm, studies comparing SST to a non-exposed control or a different type of intervention (including waitlist control) were included.

### Outcome

This review included studies reporting outcome data on the change of CMD symptoms measured by standardised tools following the intervention. Standardised tools mean they are verified for validity and reliability.

### Study type

Studies adopting either randomised trials or non-randomised studies (non-randomised controlled or non-controlled trials) were included. The author included non-randomised studies due to the anticipated limited amount of randomised trials based on the preliminary scoping search [31].

### Other characteristics

English-written, peer-reviewed studies and dissertations were included. In the protocol stage, only peer-reviewed studies were planned to be included. However, in the review stage, it was amended to include dissertations to secure the comprehensiveness of the search. Grey literature, conference abstracts, book chapters, study protocols, and case reports were excluded.

## Search method

### Data sources

A literature search was conducted in MEDLINE, Embase, PsycINFO, and Cochrane’s CENTRAL from their inception to December 13, 2022, supplemented with forward and backward citation searching.

### Search strategy

Search terms were developed by separating the main concepts (CMD and SST) and adding alternative terms and synonyms for each concept. Then, the two key concepts were combined using the Boolean operator "AND." No search filters or limits were applied. The search strategies for each database are attached in Additional file 1.

## Study selection

After removing duplicates from the initial search results, two independent reviewers (JT and NG) screened studies in two stages. In the first stage, studies were excluded if they met at least one exclusion criterion based on their titles and abstracts. Potentially relevant studies were forwarded to the second stage. In the second stage, the full text was evaluated against the eligibility criteria, and reasons for exclusion were recorded. In cases where disparities arose between the two reviewers or a consensus could not be reached, the consultation of a third reviewer (DC) was sought for resolution.

## Data extraction

Two reviewers independently extracted the following data from the finally selected studies: 1) reference, 2) eligibility criteria, 3) demographic features, 4) baseline symptoms, 5) intervention and control, 6) outcome measure, 7) study design and follow-up period, 8) sample size, 9) setting/delivering agent/intervention content, and 10) main findings.

The eligibility of outcome data is as follows. First, the eligible outcome domains were levels of depressive or anxiety symptoms. Second, all results compatible with the outcome domains were sought. There were no restrictions on the measuring time point, and all reported effect measures were collected. This less stringent standard was applied because this review aimed to synthesize data narratively, not quantitatively, based on comprehensive information.

## Risk of bias appraisal

Two reviewers independently assessed the risk of bias using Version 2 of the Cochrane risk-of-bias tool (RoB 2) and the Risk of Bias in Non-randomised Studies – of Interventions (ROBINS-I). RoB 2 was utilised for randomised trials, assessing biases in five domains: randomisation process, deviation from planned intervention, missing data, outcome measurement, and result reporting [32]. Signal questions were answered to categorize each domain's degree of bias risk as high, some concerns, or low, with the overall risk determined by a judgment for each domain. Non-randomised studies were assessed using ROBINS-I, evaluating biases in seven domains: confounding, participant selection, intervention classification, deviation from planned intervention, missing data, outcome measurement, and result reporting [33]. The degree of bias risk in each domain was classified as critical, serious, moderate, or low using signal questions. The overall risk of bias was established based on each domain's evaluation.

Two reviewers compared the planned measurements and outcomes of prespecified protocols to published literature to evaluate result reporting bias. If protocols were unavailable, the literature's "METHOD" and "RESULT" sections were compared.

The result of the risk of bias appraisal was utilised to decide which studies were included in the data synthesis, to evaluate the certainty of the evidence drawn from this review and to derive implications for enhancing research methodologies in future studies.

## Data synthesis

A narrative synthesis was adopted due to the anticipated clinical heterogeneity of included studies' characteristics (e.g., CMD concept or intervention type), which would have made a meta-analysis difficult [34]. Planned exclusion from the synthesis was non-randomised studies having a “critical” or “serious” risk of bias. The author conducted the narrative synthesis following the Popay guidelines [35]. In the preliminary synthesis phase, the author classified the studies by intervention type and tabulated their essential characteristics to identify patterns regarding the effect direction. The author then used textual descriptions to explain and interpret the findings across the studies. Finally, the author critically reflected on the findings and synthesis procedure to address the certainty of evidence.

## Results

### Search process and results

**Fig. 1:**
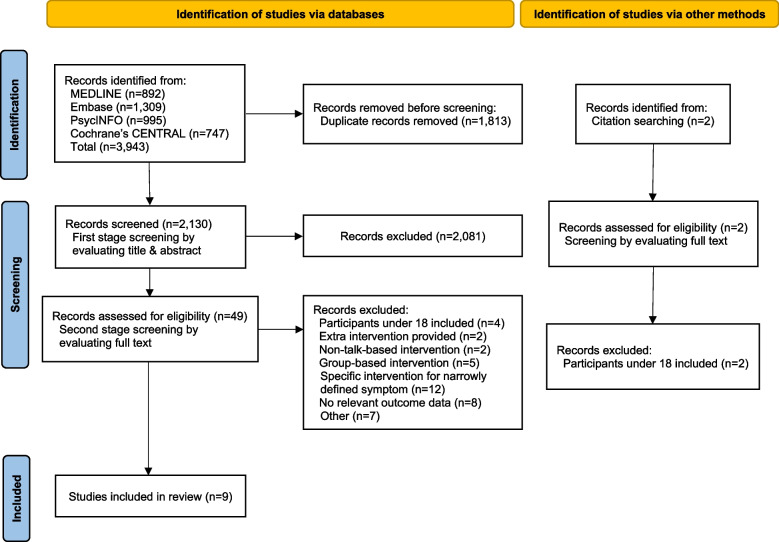
PRISMA 2020 flow diagram about the procedure of selecting studies

After removing duplicates, 2,130 studies were screened. Of these, 49 were included in the full-text assessment, and 40 were excluded. The reasons for exclusion are presented in the PRISMA 2020 flow diagram in Fig. 1 [28]. “Other” reasons for excluding the seven studies were as follows: 1) research not written in English [36], 2) research did not provide participants’ sociodemographic information [37–41], and 3) research conducted a follow-up analysis of the published literature [42] to explore a different hypothesis [43]. Two potentially eligible studies identified by the citation search were excluded due to having participants under 18 [42, 44]. Therefore, nine studies fulfilling all eligibility criteria were finally included [45–53]. Six were randomised trials [45–50], and three non-randomised studies [51–53]. Studies removed in the second stage screening are listed in Additional file 2.

## Risk of bias appraisal

Table 1 presents the result of the risk of bias assessment in the six randomised trials.

**Table 1:** Risk of bias in the six randomised trials evaluated with RoB 2

Authors (year)	Bias arising from the randomisation process	Bias due to deviations from intended interventions	Bias due to missing outcome data	Bias in measurement of the outcome	Bias in selection of the reported result	Overall bias
Parra et al. [45] (2019)	Some concerns	Some concerns	Low	High	Some concerns	High
READ et al. [46] (2016)	Some concerns	High	High	High	Some concerns	High
Nasrin et al. [47] (2016)	Low	Some concerns	High	High	Some concerns	High
Gawrysiak et al. [48] (2009)	Some concerns	Some concerns	Low	High	Some concerns	High
Ward-Ciesielski et al. [49] (2017)	Low	Some concerns	Low	Some concerns	Low	Some concerns
Sundstrom [50] (1993)	Some concerns	Some concerns	Low	Some concerns	Some concerns	Some concerns

One study was evaluated as at high risk of bias in the "bias due to deviations from intended interventions" domain since it did not use an intention-to-treat analysis and had enough participants excluded from the analysis to influence the outcome (of the assigned participants, about 60% in the intervention arm and 40% in the control arm were excluded from the analysis) [46].

Two studies were assessed as having a "high" risk of bias in the "bias due to missing outcome data" domain since they neither employed corrective procedures to address potential bias stemming from missing outcome data nor conducted analyses to confirm that the results were not problematically biased [46, 47]. Additionally, there were disparities in the percentages of missing outcome data between the two arms, and no information that the reasons for missingness were unrelated to the outcome.

Four out of six were evaluated as having a “high” risk of bias in the “bias in measurement of the outcome” domain since participants with knowledge of allocated intervention status self-measured the subjective outcomes (the level of depression or anxiety) without evidence that attempts were made to establish a neutral equipoise between the groups [45–48]. The other two were evaluated as not having a “high” risk of bias in this domain because they used active control [49, 50].

Table 2 presents the result of the risk of bias assessment in the three non-randomised studies. Two studies were assessed as having a “critical” risk in the “bias due to confounding” domain since they had no control group and did not adjust for confounders without accounting for time trends [51, 53]. In this case, it is difficult to ascertain whether pre- and post-intervention differences are attributable to the intervention or other variables. The other study was evaluated as not having a “critical” risk of bias in this domain due to its use of appropriate statistical methods to account for some confounders and time trends.

**Table 2:** Risk of bias in the three non-randomised studies evaluated with ROBINS-I

Authors (year)	Bias due to confounding	Bias in selection of participants	Bias in classification of interventions	Bias due to deviations from intended interventions	Bias due to missing data	Bias in measurement of outcomes	Bias in selection of the reported result	Overall bias
du Pont [51] (2021)	Critical	Low	Low	Serious	Serious	Serious	Moderate	Critical
Ewen et al. [52] (2018)	Serious	Low	Low	Serious	Low	Serious	Moderate	Serious
Greenberg [53] (2015)	Critical	Low	Low	Serious	Serious	Serious	Moderate	Critical

## Characteristics of the studies

Nine studies were identified [45–53], but only six randomised trials were included in the narrative synthesis. Three non-randomised studies [51–53] with a "critical" or "serious" risk of bias were excluded.

Table 3 presents the key characteristics of the six studies. They were grouped into three categories according to the intervention type. Four studies assessed behavioural activation (BA) [45–48], one assessed dialectical behaviour therapy (DBT) [49], and one assessed solution-focused psychotherapy [50]. Five were peer-reviewed studies, and one was a PhD dissertation [50]. Based on the World Bank classification [54], five studies were undertaken in high-income countries (Australia = 1, United Kingdom (UK) = 1, United States (US) = 3), and one in an upper-middle-income country (Colombia = 1). A total of 298 participants were included in six studies, with sample sizes ranging from 29 [46] to 93 [49]. The information on the sample size, along with the demographic feature of participants and baseline symptoms, are summarised in an extended table (see Additional file 3: Table S1). Data extraction results for excluded studies are presented in Additional file 4: Table S2 A, B, and C.

**Table 3:** Key characteristics of the six randomised trials

Reference	Eligibility criteria	Intervention and Control	Outcome measure^a^	Design and Follow-up	Main findings^b^
Behavioural activation					
Parra et al. [45] (2019) Colombia	Inclusion: Psychology students aged 18 or more who scored 36 or more on the SDS Exclusion: - Participants who received psychological or pharmacological intervention - Participants who used psychoactive substances	Intervention: Single-session behavioural activation Control: Waitlist control	SDS	Design: RCT Follow-up: 2, 4 weeks	There was a significant difference between groups at 2-week follow-up, but not at 4-week follow-up - SDS at 2 weeks: U Mann–Whitney = 311.000 (*p* = .03), Cohen's *d* = 0.74 - SDS at 4 weeks: U Mann–Whitney = 363.000 (*p* = .19)
READ et al. [46] (2016) Australia	Inclusion: Family/Non-family carers who met the carer criteria of the Carers Recognition Act 2004 Exclusion: Participants with depressive episodes	Intervention: Single-session behavioural activation Control: Waitlist control	DASS-21 - DASS-Depression - DASS-Anxiety	Design: RCT Follow-up: 2 weeks	1. There were no significant group x time effects in DASS-Depression and DASS-Anxiety, indicating no significant intervention effect in improving depression and anxiety - DASS-Depression: Group x time (F) = 0.72, Partial eta-squared = 0.06 - DASS-Anxiety: Group x time (F) = 0.98, Partial eta-sqaured = 0.06 2. Adherence rate^c^ - 56%
Nasrin et al. [47] (2016) United Kingdom	Inclusion: Primary care counselling services attendees aged 18 to 60 who met major depressive disorder diagnostic criteria (using SCID-I) and who scored above 10 on the PHQ-9 Exclusion: - Participants who received regular psychological intervention - Participants with particular mental conditions (e.g., self-harm, psychosis, mania, eating disorder)	Intervention: Single-session behavioural activation Control: Waitlist control	PHQ-9	Design: RCT Follow-up: 1 week	1. There was a significant difference between groups - PHQ-9: Group (F) = 16.03 (*p* = .000), Eta-sqaured = 0.30 2. Adherence rate - 51.4%
Gawrysiak et al. [48] (2009) United States	Inclusion: Introductory psychology students aged 18 or more who scored 14 or more on the BDI-II Exclusion: - Participants who received psychological or pharmacological intervention for depression - Participants who had received psychotherapy within the last 2 years - Participants with high suicide risk, psychotic symptoms, or bipolar disorder	Intervention: Single-session behavioural activation Control: No-treatment control	BDI-II BAI	Design: RCT Follow-up: 2 weeks	1. There was a significant group x time effect in BDI-II, but not in BAI - BDI-II: Group x time (F) = 12.54 (*p* < .01), Cohen's *d* = 1.61 - BAI: Group x time (F) = 1.42 (*p* = .25), Cohen's *d* = 0.36 2. Adherence rate - 72%
Dialectical behaviour therapy					
Ward-Ciesielski et al. [49] (2017) United States	Inclusion: Participants aged 18 or more who scored 10 or more on the SSI Exclusion: - Participants who received psychiatric intervention during the 1 month before the screening - Participants with severe cognitive impairment	Intervention: Single-session dialectical behaviour therapy-brief suicide intervention Control: Single-session relaxation training	PHQ-9 BAI	Design: RCT (Active control) Follow-up: 1, 4, 12 weeks	There was a significant main effect of time in PHQ and BAI, but when it comes to the condition and time-by-condition, the main effect was not significant. It means that both conditions improved those symptoms, but the evidence of a difference in effect between them did not exist - PHQ-9: Time (F) = 7.68 (*p* < .05), Condition (F) = 1.05, Condition x Time (F) = 0.16, Cohen's *d* = 0.61 (baseline to 12wk) - BAI: Time (F) = 10.41 (*p* < .05), Condition (F) = 1.05, Condition x Time (F) = 0.34, Cohen's *d* = 0.59 (baseline to 12wk)
Solution-focused psychotherapy					
Sundstrom [50] (1993) United States	Inclusion: Female college students who scored 10 to 29 on the BDI Exclusion: Participants who received psychological or pharmacological intervention for depression	Intervention: Single-session solution-focused psychotherapy Control: Single-session problem-focused psychotherapy	BDI DACL	Design: RCT (Active control) Follow-up: 7–10 days	Both single-session therapies showed a significant change in BDI and DACL, but there were no significant differences in changing BDI and DACL between the two therapies - BDI: Time (F) = 34.32 (*p* = .0001), Treatment (F) = 0.01 (*p* = .9299), Time x Treatment (F) = 0.18 (*p* = .8345), Cohen's *d* = 1.05 - DACL: Time (F) = 31.07 (*p* = .0001), Treatment (F) = 1.81 (*p* = .1872), Time x Treatment (F) = 0.45 (*p* = .6403), Cohen's *d* = 0.69

*SDS* Zung Self-Rating Depression Scale, *RCT* Randomised Controlled Trial, *DASS-21* Depression Anxiety Stress Scales 21, *DASS-Depression* Depression Anxiety Stress Scales-Depression subscale, *DASS-Anxiety* Depression Anxiety Stress Scales-Anxiety subscale, *SCID-I* Structured Clinical Interview for DSM-IV Axis 1, *PHQ-9* Patient Health Questionnaire-9, *BDI-II* Beck Depression Inventory-II, *BAI* Beck Anxiety Inventory, *SSI* Scale for Suicidal Ideation, *BDI* Beck Depression Inventory, *DACL* Depression Adjective Checklist

^a^ The outcome measures related to common mental disorders were presented

^b^ The main findings related to the outcomes of common mental disorders were summarised. The reported effect sizes and *p*-values were presented

^c^ In all studies assessing behavioural activation, the adherence rate to activity goals was calculated by the number of completed activities divided by the number of allocated activities

### Participants

The mean age of participants included in the analysis ranged from 18.40 to 52.80, with an average of 30.48. Female participants outnumbered male participants in most studies. In one study conducted in the UK [47] and two in the US [48, 49], most participants were White/Caucasian. Other studies did not provide information on ethnicity. Four studies recruited participants from a relatively homogenous population: three studies from college students [45, 48, 50] and one from family/non-family carers [46].

Regarding the eligibility criteria for the CMD symptoms, one study focusing on improving well-being to prevent depression excluded individuals having depressive episodes [46]. The remaining five studies recruited participants who scored above a threshold on standardised measures.

Regarding the baseline depressive symptoms, one study using Zung Self-Rating Depression Scale (SDS) reported that the baseline levels were subclinical or mild [45]. In the study that excluded individuals with depressive episodes, the baseline levels were normal or mild based on Depression Anxiety Stress Scales 21 (DASS-21) [46]. Two studies employed Patient Health Questionnaire-9 (PHQ-9), and the baseline level in both was moderately severe [47, 49]. One study employed Beck Depression Inventory-II (BDI-II), and the baseline level was mild or moderate [48]. One study employed Beck Depression Inventory (BDI) and Depression Adjective Checklist (DACL), and the baseline level was mild to moderate based on BDI [50]. Regarding the baseline anxiety symptoms, three studies measured it using the DASS-Anxiety subscale [46] and the Beck Anxiety Inventory (BAI) [48, 49]. The baseline level was normal in one study [46] and mild to moderate in the other [48, 49]. In short, six studies targeted participants with non-severe depressive symptoms with or without anxiety. The baseline level of each symptom was estimated based on the literature on each assessment tool [55–58].

### Intervention and control

Three intervention types were identified: BA, DBT, and solution-focused psychotherapy. Regarding the control, three of four BA evaluating studies adopted the waitlist control, while the other “no treatment” [48]. Studies on DBT and solution-focused psychotherapy adopted relaxation training and problem-focused psychotherapy as an active control, respectively [49, 50]. All interventions were delivered in person.

Table 4 contains descriptions of each intervention in detail. One of four BA assessment studies self-developed a single-session BA protocol [48]. It reduced the number of sessions from nine to one by using a non-gradual approach to activity scheduling. In contrast to the traditional gradual approach, this method immediately targets a greater number of behaviours for activation. All four studies evaluating BA employed this protocol. The study on DBT used a self-developed protocol based on existing single-session intervention [59]. The study of solution-focused psychotherapy did not specify the source of the treatment modality. The duration of BA, DBT, and solution-focused psychotherapy sessions were 90, 45–60, and 40–50 min, respectively.

**Table 4:** Description of the interventions adopted in the six randomised trials

Reference	Setting	Delivering agent	Intervention content
Behavioural activation			
Parra et al. [45] (2019)	Country: Colombia Platform^a^: Not clearly indicated	Psychology professionals/Clinical psychology specialists - Trained via attending a behavioural activation training course	Duration: 90 min Content: - Presenting information about depression, well-being, and a rationale for behavioural activation - Identifying key life values and relevant activity goals - Discuss how to monitor, review, and modify activity goals - Discuss how to troubleshoot obstacles to achieving activity goals
READ et al. [46] (2016)	Country: Australia Platform: Curtin University Psychology Clinic	Clinical psychology postgraduate students - Trained via educational DVD and senior clinical psychologist supervision	Duration: 90 min Content: Same as above Materials: A workbook summarizing the intervention was provided
Nasrin et al. [47] (2016)	Country: United Kingdom Platform: Not clearly indicated	Trained clinical psychologists	Duration: 90 min Content: Same as above
Gawrysiak et al. [48] (2009)	Country: United States Platform: Not clearly indicated	Trained clinical psychology doctoral students	Duration: 90 min Content: Same as above
Dialectical behaviour therapy			
Ward-Ciesielski et al. [49] (2017)	Country: United States Platform: University outpatient clinic	DBT-trained masters'-level therapists	Duration: 45–60 min Content: - Presenting 5 DBT skills (Distraction, Opposite-to-emotion action, Mindfulness, Mindfulness of current emotion, Changing your body chemistry) - Explaining and practising the skills Materials: Individualized information about mental health resources was provided at the end of the session
Solution-focused psychotherapy			
Sundstrom [50] (1993)	Country: United States Platform: University psychology building	Female counsellors (counselling psychology postgraduate students, counselling psychology interns, professionals) - All received a 2-h training	Duration: 40–50 min Content: - Aiming to direct clients' attention from pessimistic rumination to optimistic methods to make changes - Aiming to assist clients in taking control of their problems - Focusing on coping strategies that have proven effective and clients' resources - Providing feedback, including compliments on clients' resources

*DBT* Dialectical behaviour therapy

^a^ Platform refers to the location where each intervention is implemented

The included techniques emphasised practical coping skills, unlike traditional counseling methods focusing on symptom exploration and venting [50]. BA sets activity goals linked to essential life values, enabling participants to cope with depression by practising them [48]. The DBT introduces five skills (e.g., mindfulness) and trains participants to utilise them when emotional difficulties arise [49]. Solution-focused psychotherapy focuses on the clients’ positive coping strategy and personal resources to assist them in managing their difficulties independently [50].

The intervention providers were all trained mental health professionals. Three studies mentioned the platform where the intervention was administered; all were places affiliated with universities [46, 49, 50]. No related information was found in the other three.

### Outcome

Three of four BA investigating studies found statistically significant intervention effects in improving depression [45, 47, 48], while one did not [46]. Eligibility criteria differed between studies with and without significant findings. Three studies with positive findings included participants scoring over the threshold measured by the standardised depression scale, while the study with non-significant findings excluded participants with depressive episodes. However, not all positive-result studies had considerably greater depression levels at baseline than that with non-significant results. The participants in the study with no significant result had normal or mild depression at baseline. Among the trials with positive outcomes, one had participants with moderately severe depression [47], while the other two had participants with mild (or subclinical) and moderate depression, respectively [45, 48]. Regarding the adherence rate to activity goals, the study with non-significant effects had a 56% adherence rate. Two positive-result studies had 51.4% and 72%, respectively [47, 48], while the other did not provide information on adherence [45]. The follow-up period was one to two weeks after the intervention across the studies. Only one positive-result study followed participants for four weeks beyond the two weeks, reporting a statistically significant effect at two weeks but not at four weeks [45]. The two studies evaluated the effect on anxiety, but it was not statistically significant [46, 48]. Both studies had participants with normal and mild to moderate anxiety at baseline, respectively.

The study assessing DBT reported a significant intervention effect on depression and anxiety at one, four, and twelve weeks after the intervention [49]. However, there was no significant difference in the effect on those outcomes compared to the control (relaxation training). The participants had moderately severe depression and mild to moderate anxiety at baseline. The study evaluating solution-focused psychotherapy reported a significant effect on depression at seven to ten days after the intervention [50]. However, there was also no significant difference in the effect compared to the control (problem-focused psychotherapy). The participants had mild to moderate depression.

In summary, five of six studies reported that SST was effective in improving depression, whereas only one study showed its effectiveness in mitigating anxiety. In other words, the clinical effectiveness of SST appears to be favourable, in the short-term treatment for non-severe depressive symptoms. However, more than half of the studies included in the synthesis had a “high” risk of bias, and some had small sample sizes. In addition, the total amount of studies was small, and the amount for each intervention type was even smaller. These factors render this evidence uncertain.

## Discussion

This systematic review evaluated the clinical effectiveness of SST on CMD symptoms in adults. Five of the six randomised trials included in the synthesis reported favourable results in improving depression. This positive finding aligns with previous literature reviews that addressed SST as a promising intervention [18, 24]. In addition, although the target participants or symptoms are somewhat different, this result is consistent with previous systematic reviews in that positive intervention effects were observed in improving mental health conditions [25, 26].

However, some characteristics of the included studies make this evidence less certain. First, the number of studies was insufficient. Six studies were included in the synthesis; four evaluated BA, and only one each evaluated DBT and solution-focused psychotherapy. Second, there is concern about bias. Four of six were rated to have a “high” risk of bias overall. Third, some studies have insufficient sample sizes to detect statistical findings. Two studies with non-significant effects discussed the small sample size as their limitation [46, 48]. In these studies, whether the statistical insignificance results from the small sample size or the ineffective intervention is unclear.

The review process also causes uncertainty. This review included only English-written studies and excluded grey literature. This restriction may have hindered the comprehensive inclusion of relevant studies. However, the robust systematic review methodology the author adopted complements these limitations. Two reviewers independently conducted the literature screening, data extraction, and bias appraisal, crosschecking the discrepancy. In addition, this review conducted the narrative synthesis following the guideline and adhered to PRISMA 2020 statement. Therefore, despite the abovementioned limitations, the author believes this review includes representative relevant studies and that the findings can have implications.

This review suggests research topics that could promote the widespread use of SST, one of which is exploring the characteristics of those who can benefit from it. Although integration of SST into ongoing services may decrease the treatment gap, there are concerns that symptoms may worsen when SST is delivered to unsuitable clients. Its effectiveness may vary depending on the individual’s characteristics [24]. By identifying the characteristics suitable for SST, its potential can be maximized. Given the comprehensive concept of SST, suitable characteristics may differ depending on the intervention type. Nevertheless, since there are common aspects identified in this review (e.g., to inform practical coping methods), there would be shared features that can benefit from those common aspects.

The severity of symptoms is a trait that needs to be studied. Qualitative interviews found that the difference in accessibility between walk-in single-session counselling and traditional counselling was the most significant factor in explaining why walk-in model users recovered from CMD symptoms faster [42]. In addition, walk-in model users expressed satisfaction with the therapists' practical feedback [42]. These findings suggest that individuals who are most likely to benefit from SST are those who can actively engage in the services and utilise the coping methods learned. In other words, individuals with preserved executive function, a set of cognitive processes that allow people to plan and control their behaviour to achieve goals [60], may be more likely to benefit from SST. Research has shown that the severity of CMD symptoms is correlated with the degree of executive dysfunction [61–63], suggesting SST may not benefit people whose CMD symptoms are severe enough to impair their executive function. This idea is consistent with the previous literature indicating that SST may be most helpful for less distressed people [24]. In addition, the overall positive outcomes of the studies in this review may be preliminary evidence supporting this notion, given that all studies had participants with non-severe depression at baseline.

The client's motivational level also should be considered. Prior research suggests that individuals with high motivation to change may experience clinical benefits from SST [24], implying that those with low motivation may be unsuitable for SST. As stated in the “BACKGROUND” section, individuals in low-resource settings believe that mental health treatment is futile for their emotional difficulties unless socioeconomic adversity is addressed [13]. They may lack the motivation to improve their symptoms through treatment, suggesting that CMD symptoms associated with adverse social circumstances may not be appropriate for SST. This notion is supported by the study showing that walk-in single-session counselling users with domestic violence or housing/financial issues recovered from CMD symptoms more slowly than those without such problems [42]. In sum, individuals with non-severe symptoms and high motivation to change are likely to experience clinical benefits from SST. However, since this estimation is based on assumptions and methodologically weak studies, research with more methodological rigour is needed to clarify this preliminary notion.

The following are further research topics that need exploration to facilitate the widespread use of SST. First, longer follow-up studies are needed to assess its long-term effects. Most included studies had a follow-up period of within one month, and four of six had within two weeks. Due to the fluctuating nature of CMD symptoms over time and their potential for recurrence, evidence solely derived from short-term follow-up studies cannot definitively support the clinical utility of SST. Second, there is a need to investigate the clinical effectiveness of SST delivered by non-professionals. The effect of psychological intervention delivered by lay health workers on CMDs has evidence [64]. However, according to this review’s findings, few studies have evaluated whether SST delivered by non-professionals would be effective on CMDs. The high global prevalence of CMDs and the complex factors that hinder access to services, such as stigma, make it challenging to meet the demand for treatment with only experts. Generating evidence for the non-professionals-delivered SST will contribute to reducing the treatment gap by expanding its applicability to diverse settings. Third, since several randomised trials evaluating single-session BA were identified, future research should consider meta-analysis. For SST to be implemented and scaled up, stakeholders (e.g., policymakers) should understand its clinical value. Meta-analysis can effectively disseminate knowledge about clinical utility by generating clear quantitative evidence. Fourthly, this review indicates that SST is less effective for anxiety than depression. Given that only three interview types were assessed in this review (BA, DBT, and solution-focused psychotherapy), there is a need for additional research to investigate single-session intervention techniques that show greater effectiveness in addressing anxiety.

The methodological issues to be addressed in future studies are as follows. First, future trials should consider adopting outcome measurement methods other than self-reporting. All included studies measured the outcomes in a self-report manner. CMD symptom assessment can be influenced by subjectivity, and the participants would have been aware of the allocated status due to the nature of the intervention. Under these conditions, self-reporting methods increase the risk of bias in the outcome measurement domain. This bias can diminish when blinded evaluators measure outcomes using observer-administered instruments like Montgomery-Asberg Depression Rating Scale (MADRS) [65]. Second, future research should follow the reporting guidelines (e.g., CONSORT 2010 statement [66]) so that the risk of bias can be assessed with more clarity. Third, sufficient sample sizes should be adopted to ensure the precision of the results.

## Conclusion

This systematic review can provide preliminary evidence that SST may be effective for reducing CMD symptoms, particularly depression. However, it cannot reach a definitive conclusion due to the limitations described previously. This review suggests various research topics necessary to facilitate the implementation and scaling-up of SST. In addition, this review highlights methodological issues that should be addressed in future trials.

### Supplementary Information

**Additional file 1.** Search strategies for each database.

**Additional file 2.** Studies excluded in second stage screening.

**Additional file 3.** Extended characteristics of the six randomised trials.

**Additional file 4**. Data extraction results for the excluded three non-randomised studies

## Data Availability

All data generated or analysed during this study are included in this published article and its supplementary information files.

## Abbreviations

CMD: Common Mental Disorder
DMS-5: Diagnostic and Statistical Manual of Mental Disorders, Fifth Edition
YLD: Years Lived with Disability
SST: Single-Session Therapy
PROSPERO: International Prospective Register of Systematic Reviews
PRISMA: Preferred Reporting Items for Systematic Reviews and Meta-Analyses
RoB 2: Version 2 of the Cochrane risk-of-bias tool
ROBINS-I: Risk of Bias in Non-randomised Studies Of Interventions
BA: Behavioural Activation
DBT: Dialectical Behaviour Therapy
UK: United Kingdom
US: United States
SDS: Zung Self-Rating Depression Scale
DASS-21: Depression Anxiety Stress Scales 21
PHQ-9: Patient Health Questionnaire-9
BDI- II: Beck Depression Inventory-II
BDI: Beck Depression Inventory
DACL: Depression Adjective Checklist
BAI: Beck Anxiety Inventory
MADRS: Montgomery-Asberg Depression Rating Scale
CONSORT: Consolidated Standards of Reporting Trials
