# Supplementary material for: Effectiveness of single-session therapy for adult common mental disorders: a systematic review
Source: BMC Psychol. 2023 Nov 7;11:373. doi: 10.1186/s40359-023-01410-0 (PMC10631081; doi:10.1186/s40359-023-01410-0)
Supplement: Supplementary file 1 — Additional file 1. Search strategies for each database. [file 40359_2023_1410_MOESM1_ESM.docx]

**Additional file 1** (.docx) Search strategies for each database

**MEDLINE Search Strategy**

**MEDLINE was searched via the OVID interface on December 13, 2022. The database coverage was from 1946 to December 12, 2022.**

1. mental health.mp. or exp Mental Health/

2. exp Mental Disorders/ or mental disorder*.mp.

3. exp Depression/

4. depression.mp.

5. depressed.mp.

6. depressive.mp.

7. 3 or 4 or 5 or 6

8. exp Anxiety/

9. anxiety.mp.

10. anxious.mp.

11. 8 or 9 or 10

12. 1 or 2 or 7 or 11

13. single session*.mp.

14. therap*.mp.

15. consult*.mp.

16. intervention*.mp. or exp Psychosocial Intervention/

17. exp Counseling/ or counsel*.mp.

18. exp Psychotherapy/ or psychotherap*.mp.

19. walk-in.mp.

20. social work.mp. or exp Social Work/

21. 14 or 15 or 16 or 17 or 18 or 19 or 20

22. 13 and 21

23. one-off consultation*.mp.

24. one session treatment*.mp.

25. 22 or 23 or 24

26. 12 and 25

**Embase Search Strategy**

**Embase was searched via the OVID interface on December 13, 2022. The database coverage was from 1974 to December 12, 2022.**

1. mental health.mp. or exp mental health/

2. exp mental disease/ or mental disease*.mp.

3. exp depression/

4. depression.mp.

5. depressed.mp.

6. depressive.mp.

7. 3 or 4 or 5 or 6

8. exp anxiety disorder/ or exp anxiety/

9. anxiety.mp.

10. anxious.mp.

11. 8 or 9 or 10

12. 1 or 2 or 7 or 11

13. single session*.mp.

14. therap*.mp.

15. consult*.mp. or exp consultation/

16. intervention*.mp. or exp psychosocial intervention/

17. exp counseling/ or counsel*.mp.

18. exp psychotherapy/ or psychotherap*.mp.

19. walk-in.mp.

20. social work.mp. or exp social work/

21. 14 or 15 or 16 or 17 or 18 or 19 or 20

22. 13 and 21

23. one-off consultation*.mp.

24. one session treatment*.mp.

25. 22 or 23 or 24

26. 12 and 25

**PsycINFO Search Strategy**

**PsycINFO was searched via the OVID interface on December 13, 2022. The database coverage was from 1806 to December week 1, 2022.**

1. mental health.mp. or exp Mental Health/

2. exp Mental Disorders/ or mental disorder*.mp.

3. exp "Depression (Emotion)"/ or exp Major Depression/

4. depression.mp.

5. depressed.mp.

6. depressive.mp.

7. 3 or 4 or 5 or 6

8. exp Anxiety Disorders/ or exp Anxiety/

9. anxiety.mp.

10. anxious.mp.

11. 8 or 9 or 10

12. 1 or 2 or 7 or 11

13. single session*.mp.

14. therap*.mp.

15. consult*.mp.

16. intervention*.mp. or exp Intervention/

17. exp Counseling/ or counsel*.mp.

18. exp Psychotherapy/ or psychotherap*.mp.

19. exp Walk In Clinics/ or walk-in.mp.

20. social work.mp.

21. 14 or 15 or 16 or 17 or 18 or 19 or 20

22. 13 and 21

23. one-off consultation*.mp.

24. one session treatment*.mp.

25. 22 or 23 or 24

26. 12 and 25

**Cochrane’s CENTRAL Search Strategy**

**Cochrane’s CENTRAL was searched on December 13, 2022, using the advanced search interface. The database coverage was not identified.**

1. MeSH descriptor: [Mental Health] explode all trees

2. ("mental health"):ti,ab,kw

3. #1 OR #2

4. MeSH descriptor: [Mental Disorders] explode all trees

5. (mental NEXT disorder*):ti,ab,kw

6. #4 OR #5

7. MeSH descriptor: [Depression] explode all trees

8. (depression):ti,ab,kw

9. (depressed):ti,ab,kw

10. (depressive):ti,ab,kw

11. #7 OR #8 OR #9 OR #10

12. MeSH descriptor: [Anxiety] explode all trees

13. (anxiety):ti,ab,kw

14. (anxious):ti,ab,kw

15. #12 OR #13 OR #14

16. #3 OR #6 OR #11 OR #15

17. (single NEXT session*):ti,ab,kw

18. (therap*):ti,ab,kw

19. (consult*):ti,ab,kw

20. MeSH descriptor: [Psychosocial Intervention] explode all trees

21. (intervention*):ti,ab,kw

22. #20 OR #21

23. MeSH descriptor: [Counseling] explode all trees

24. (counsel*):ti,ab,kw

25. #23 OR #24

26. MeSH descriptor: [Psychotherapy] explode all trees

27. (psychotherap*):ti,ab,kw

28. #26 OR #27

29. ("walk-in"):ti,ab,kw

30. MeSH descriptor: [Social Work] explode all trees

31. ("social work"):ti,ab,kw

32. #30 OR #31

33. #18 OR #19 OR #22 OR #25 OR #28 OR #29 OR #32

34. #17 AND #33

35. (one-off NEXT consultation*):ti,ab,kw

36. (one NEXT session NEXT treatment*):ti,ab,kw

37. #34 OR #35 OR #36

38. #16 AND #37
