# Supplementary material for: Effectiveness of single-session therapy for adult common mental disorders: a systematic review
Source: BMC Psychol. 2023 Nov 7;11:373. doi: 10.1186/s40359-023-01410-0 (PMC10631081; doi:10.1186/s40359-023-01410-0)
Supplement: Supplementary file 2 — Additional file 2. Studies excluded in second stage screening. [file 40359_2023_1410_MOESM2_ESM.docx]

**Additional file 2** (.docx) Studies excluded in second stage screening

1. Aakhus E, Engedal K, Aspelund T, Selbaek G. Single session educational programme for caregivers of psychogeriatric in-patients--results from a randomised controlled pilot study. Int J Geriatr Psychiatry. 2009;24(3):269-74.
2. Al-khatib B, Norris S. A Family Consultation Service: Single session intervention to build the mental health and wellbeing of children and their families. Educ Child Psychol. 2015;32(4):7-20.
3. Allen CW, Siedlecki T, Nagel AG, Tan JS, Datta P, Henkler KC, et al. Brief behavioral interventions at free medical fairs. Int J Psychiatry Med. 2018;53(5-6):371-83.
4. Armento MEA, McNulty JK, Hopko DR. Behavioral activation of religious behaviors (BARB): Randomized trial with depressed college students. Psycholog Relig Spiritual. 2012;4(3):206-22.
5. Bentley KH, Boettcher H, Bullis JR, Carl JR, Conklin LR, Sauer-Zavala S, et al. Development of a Single-Session, Transdiagnostic Preventive Intervention for Young Adults at Risk for Emotional Disorders. Behav Modif. 2018;42(5):781-805.
6. Bermudez MB, Costanzi M, Macedo MJA, Tatton-Ramos T, Xavier ACM, Ferrao YA, et al. Improved quality of life and reduced depressive symptoms in medical students after a single-session intervention. Braz J Psychiatry. 2020;42(2):145-52.
7. Bullock J, Whiteley C, Moakes K, Clarke I, Riches S. Single-session Comprehend, Cope, and Connect intervention in acute and crisis psychology: A feasibility and acceptability study. Clin Psychol Psychother. 2021;28(1):219-25.
8. Cannistra F, Piccirilli F, D'Alia PP, Giannetti A, Piva L, Gobbato F, et al. Examining the incidence and clients' experiences of single session therapy in Italy: A feasibility study. Aust N Z J Fam Ther. 2020;41(3):271-82.
9. Carleton RN, Korol S, Mason JE, Hozempa K, Anderson GS, Jones NA, et al. A longitudinal assessment of the road to mental readiness training among municipal police. Cogn Behav Ther. 2018;47(6):508-28.
10. Coverley CT, Garralda ME, Bowman F. Psychiatric intervention in primary care for mothers whose schoolchildren have psychiatric disorder. Br J Gen Pract. 1995;45(394):235-7.
11. Danitz SB, Orsillo SM. The Mindful Way Through the Semester: An Investigation of the Effectiveness of an Acceptance-Based Behavioral Therapy Program on Psychological Wellness in First-Year Students. Behav Modif. 2014;38(4):549-66.
12. Dass-Brailsford P, Thomley RSH. Using walk-in counseling services after Hurricane Katrina: A program evaluation. J Aggress Maltreat Trauma. 2015;24(4):419-32.
13. Denner S, Reeves S. Single session assessment and therapy for new referrals to CMHTS. J Ment Health. 1997;6(3):275-80.
14. Duan W, Bu H, Zhao J, Guo X. Examining the mediating roles of strengths knowledge and strengths use in a 1-year single-session character strength-based cognitive intervention. J Happiness Stud. 2019;20(6):1673-88.
15. Ghafoori B, Fisher D, Korosteleva O, Hong M. A Randomized, Controlled Pilot Study of a Single-Session Psychoeducation Treatment for Urban, Culturally Diverse, Trauma-Exposed Adults. J Nerv Ment Dis. 2016;204(6):421-30.
16. Hadlandsmyth K, Dindo LN, Wajid R, Sugg SL, Zimmerman MB, Rakel BA. A single-session acceptance and commitment therapy intervention among women undergoing surgery for breast cancer: A randomized pilot trial to reduce persistent postsurgical pain. Psychooncology. 2019;28(11):2210-7.
17. Harper-Jaques S, Foucault D. Walk-in single-session therapy: Client satisfaction and clinical outcomes. J Syst Ther. 2014;33(3):29-49.
18. Harper-Jaques S, McElheran N, Slive A, Leahey M. A comparison of two approaches to the delivery of walk-in single session mental health therapy. J Syst Ther. 2008;27(4):40-53.
19. Johnson KL. Integrated psychological services in pediatric primary care: A program evaluation. Diss Abstr Int. 2022;83(4-B):No-Specified.
20. Kachor M, Brothwell J. Improving youth mental health services access using a single-session therapy approach. J Syst Ther. 2020;39(3):46-55.
21. Lamprecht H, Laydon C, McQuillan C, Wiseman S, Williams L, Gash A, et al. Single-session solution-focused brief therapy and self-harm: A pilot study. J Psychiatr Ment Health Nurs. 2007;14(6):601-2.
22. Lavelle J, Storan D, Murthy VE, De Dominicis N, Mulcahy HE, McHugh L. Brief and Telehealth Acceptance and Commitment Therapy (ACT) Interventions for Stress in Inflammatory Bowel Disease (IBD): A Series of Single Case Experimental Design (SCED) Studies. J Clin Med. 2022;11(10):2757.
23. Le Gros J, Wyder M, Brunelli V. Single session work: Implementing brief intervention as routine practice in an acute care mental health assessment service. Australas Psychiatry. 2019;27(1):21-4.
24. Luutonen S, Santalahti A, Makinen M, Vahlberg T, Rautava P. One-session cognitive behavior treatment for long-term frequent attenders in primary care: randomized controlled trial. Scand J Prim Health Care. 2019;37(1):98-104.
25. McManus F, Van Doorn K, Yiend J. Examining the effects of thought records and behavioral experiments in instigating belief change. J Behav Ther Exp Psychiatry. 2012;43(1):540-7.
26. Miller JK. Walk-in single session team therapy: A study of client satisfaction. J Syst Ther. 2008;27(3):78-94.
27. Morikawa A, Takayama M, Yoshizawa E. The efficacy of thought field therapy and its impact on heart rate variability in student counseling: A randomized controlled trial. Explore (NY). 2022;18(5):579-86.
28. Mulligan J, Olivieri H, Young K, Lin J, Anthony SJ. Single session therapy in pediatric healthcare: the value of adopting a strengths-based approach for families living with neurological disorders. Child Adolesc Psychiatry Ment Health. 2022;16(1):59.
29. Nowlan JS, Wuthrich VM, Rapee RM, Kinsella JM, Barker G. A Comparison of Single-Session Positive Reappraisal, Cognitive Restructuring and Supportive Counselling for Older Adults with Type 2 Diabetes. Cognit Ther Res. 2016;40(2):216‐29.
30. Perkins R, Scarlett G. The effectiveness of single session therapy in child and adolescent mental health. Part 2: an 18-month follow-up study. Psychol Psychother: Theory Res Pract. 2008;81(Pt 2):143-56.
31. Perkins R. The effectiveness of one session of therapy using a single-session therapy approach for children and adolescents with mental health problems. Psychol Psychother: Theory Res Pract. 2006;79(Pt 2):215-27.
32. Riemer M, Stalker CA, Dittmer L, Cait C-A, Horton S, Kermani N, et al. The walk-in counselling model of service delivery: Who benefits most? Can J Commun Ment Health. 2018;37(2):29-47.
33. Rodriguez LM, Lee KDM, Onufrak J, Dell JB, Quist M, Drake HP, et al. Effects of a brief interpersonal conflict cognitive reappraisal intervention on improvements in access to emotion regulation strategies and depressive symptoms in college students. Psychol Health. 2020;35(10):1207-27.
34. Ryan C, O'Connor S. Single session psychology clinic for parents of children with autism spectrum disorder: A feasibility study. J Child Fam Stud. 2017;26(6):1614-21.
35. Sherpa LR. The impact of acculturation and gender on how Japanese-American subjects respond to Davanloo's partial trial therapy as measured by levels of anxiety, depression, and self-esteem. Diss Abstr Int. 2001;62(1-B):564.
36. Silverman WH, Beech RP. Length of intervention and client assessed outcome. J Clin Psychol. 1984;40(2):475-80.
37. Stein AT, Shumake J, Beevers CG, Smits JAJ. Therapist Guided Activity Practice for Depressive Symptoms in University Students: a Randomized Controlled Trial. Cognit Ther Res. 2020;44(3):499‐510.
38. Van Dee V, Deschamps PKH. Less effort, more effects? Single-session interventions for psychiatric problems in children. Tijdschr Psychiatr. 2017;59(7):441-2.
39. Vera MF, Cardenas SJ, Vera JG. Effects of a single-session intervention on anxiety and depression in informal primary caregivers. Efectos de una intervencion de sesion unica sobre la ansiedad y depresion en cuidadores primarios informales. 2016;26(1):69-80.
40. Williams AS, Sougleris C, Howell C. How acceptable are one-off consultations for consumers? Further information on Item 291. Australas Psychiatry. 2011;19(1):70-3.
